# μ-Bromido-bis{μ-2,2′-[4,7-diaza­decane-1,10-diylbis(nitrilo­methanylyl­idene)]diphenolato}tricopper(II) bromide dimethyl­formamide disolvate

**DOI:** 10.1107/S160053681103090X

**Published:** 2011-08-06

**Authors:** Gervas Assey, Ray J. Butcher, Yilma Gultneh

**Affiliations:** a Department of Chemistry, Howard University, 525 College Street NW, Washington, DC 20059, USA

## Abstract

The complex mol­ecule of the title compound, [Cu_3_Br(C_22_H_28_N_4_O_2_)_2_]Br·2C_3_H_7_NO, contains three copper atoms, two of which are five-coordinate within a square-pyramidal environment and linked by a bridging Br atom occupying the apical position in each square pyramid. The remaining Cu atom is four-coordinate but with considerable tetra­hedral disortion [the dihedral angle between the two chelate planes is 69.21 (7)°]. There are two mol­ecules of dimethyl­formamide (DMF) present as solvent mol­ecules, one of which is disordered over two equivalent conformations with occupancies of 0.603 (5) and 0.397 (5). The amine H atoms are involved in both inter- and intra­molecular hydrogen-bonding inter­actions with the Br and O atoms of the cation, as well as with the O atom of the ordered DMF mol­ecule.

## Related literature

For information concerning the τ parameter, see: Addison *et al.* (1984 ▶). For background to the use of multi-nuclear copper complexes in metallo-enzymes for catalyzing the four-electron reduction of oxygen to water, see: Yoon *et al.* (2005 ▶); Solomon *et al.* (1996 ▶); Mukherjee *et al.* (2003 ▶); Mirica & Stack (2005 ▶); Augustine *et al.* (2010 ▶); Chaudhuri *et al.* (1992 ▶). For general background to multinuclear copper types, see: Miessler & Tarr (2005 ▶); Mukherjee *et al.* (2003 ▶); Chen *et al.* (2010 ▶); Lawton *et al.* (2009 ▶); Hakulinen *et al.* (2008 ▶). For information concerning the Type II and Type III site: Pompidor *et al.* (2008 ▶); Li *et al.* (2009 ▶). For model studies of the multi-copper site: Cole *et al.* (1996 ▶); Kataoka *et al.* (2009 ▶); Paine *et al.* (2004 ▶).
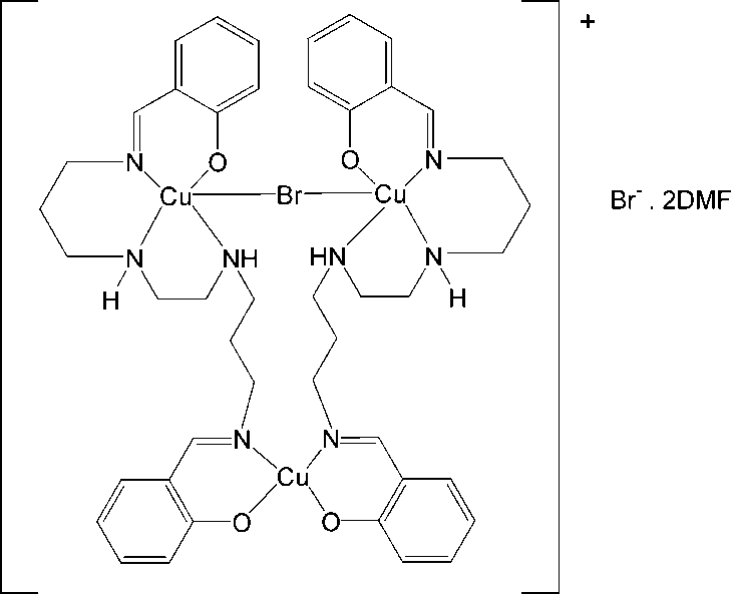


## Experimental

### 

#### Crystal data

[Cu_3_Br(C_22_H_28_N_4_O_2_)_2_]Br·2C_3_H_7_NO
*M* *_r_* = 1257.60
Hexagonal, 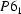

*a* = 18.7450 (2) Å
*c* = 28.2531 (5) Å
*V* = 8597.4 (2) Å^3^
*Z* = 6
Mo *K*α radiation
μ = 2.55 mm^−1^
*T* = 200 K
0.51 × 0.42 × 0.38 mm

#### Data collection

Oxford Diffraction Gemini R diffractometer
Absorption correction: multi-scan (*CrysAlis RED*; Oxford Diffraction, 2009 ▶) *T*_min_ = 0.782, *T*_max_ = 1.000
95983 measured reflections
11660 independent reflections
8463 reflections with *I* > 2σ(*I*)
*R*_int_ = 0.070

#### Refinement

*R*[*F* ^2^ > 2σ(*F* ^2^)] = 0.030
*wR*(*F* ^2^) = 0.062
*S* = 0.91
11660 reflections
662 parameters
85 restraints
H-atom parameters constrained
Δρ_max_ = 0.39 e Å^−3^
Δρ_min_ = −0.26 e Å^−3^
Absolute structure: Flack (1983 ▶), 5710 Friedel pairs
Flack parameter: 0.007 (5)

### 

Data collection: *CrysAlis CCD* (Oxford Diffraction, 2009 ▶); cell refinement: *CrysAlis RED* (Oxford Diffraction, 2009 ▶); data reduction: *CrysAlis RED*; program(s) used to solve structure: *SHELXS97* (Sheldrick, 2008 ▶); program(s) used to refine structure: *SHELXL97* (Sheldrick, 2008 ▶); molecular graphics: *SHELXTL* (Sheldrick, 2008 ▶); software used to prepare material for publication: *SHELXTL*.

## Supplementary Material

Crystal structure: contains datablock(s) I, global. DOI: 10.1107/S160053681103090X/bt5585sup1.cif

Structure factors: contains datablock(s) I. DOI: 10.1107/S160053681103090X/bt5585Isup2.hkl

Additional supplementary materials:  crystallographic information; 3D view; checkCIF report

## Figures and Tables

**Table 1:** Hydrogen-bond geometry (Å, °)

*D*—H⋯*A*	*D*—H	H⋯*A*	*D*⋯*A*	*D*—H⋯*A*
N2*A*—H2*AB*⋯O2*B*	0.93	2.00	2.919 (4)	170
N3*A*—H3*AB*⋯Br2	0.93	2.50	3.429 (3)	174
N2*B*—H2*BB*⋯O2*A*	0.93	2.14	3.061 (4)	172
N3*B*—H3*BB*⋯O1*S*	0.93	2.39	3.005 (4)	123

**Fig. 1.**
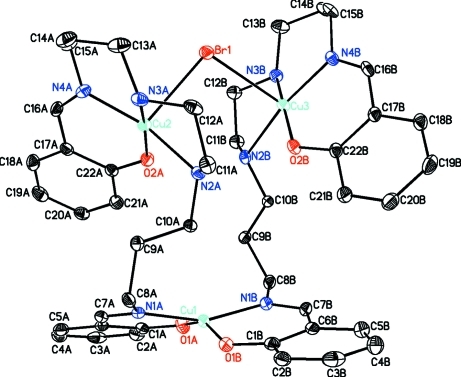
Diagram of trinuclear Cu cation, C47H63BrCu3N9O5 showing atom labeling. All H atoms are removed for clarity.

**Fig. 2.**
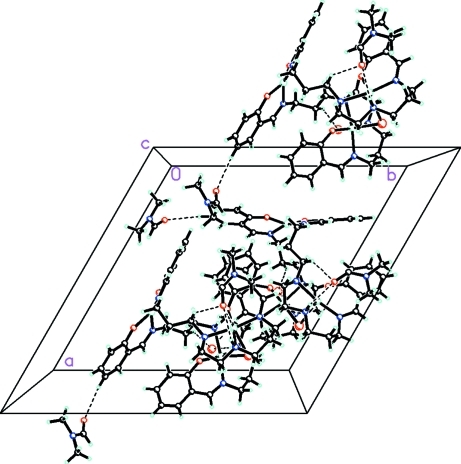
The molecular packing for C50H70Br2Cu3N10O6 viewed down the c axis. Hydrogen bonds are shown by dashed lines.

## supplementary crystallographic information

### Comment

Copper is one of the late transition metals that are essential to the life of animals and plants (Miessler & Tarr, 2005). The metal is present in a number of enzymes such as tyrosinase, amine oxidase, laccase, ascorbate oxidase, ceruloplasmin, superoxide dismutase, nitrile reductase and plastocyanin (Miessler & Tarr, 2005). There are three types of copper atoms present in enzymes, namely Type (I), Type (II) and Type (III). Type (I) copper is responsible for the blue color of blue oxidases and shows an intense visible absorption band near 600 nm (Miessler & Tarr, 2005; Mukherjee *et al.* 2003), while Type II contains normal tetragonally distorted Cu(II) (Pompidor *et al.* 2008). Type III contains two strongly antiferromagnetically coupled Cu(II) centers and is diagmagetic (Li *et al.* 2009). The role of Type (I) copper is to transfer electrons to the tri-nuclear unit in ascorbate oxidase for a simultaneous four-electron reduction of dioxygen (O_2_) (Yoon *et al.* 2005). The tri-nuclear cluster of the enzymes is comprised of a Type (II) copper center and a Type (III) coupled binuclear site in relatively close proximity where oxygen reduction is effected (Yoon *et al.* 2005; Solomon *et al.* 1996; Chen *et al.* 2010; Lawton *et al.* 2009; Hakulinen *et al.*, 2008). Generally, the multinuclear copper complexes that are in the group of metalloenzymes are used to catalyze the four-electron reduction of O_2_ to H_2_O (Yoon *et al.* 2005; Mukherjee *et al.* 2003; Mirica *et al.* 2005; Augustine *et al.* 2010; Chaudhuri *et al.* 1992; Chen *et al.* 2010).

A number of researchers have synthesized or studied model complexes of multicopper oxidases. Paine *et al.* 2004 synthesized and characterized seven copper (II) complexes with a ligand 2,2'-selonobis(4,6-di-*tert*-butylphenol) (H_2_L). Two of the complexes were found to belong to the asymmetric tri-nuclear copper (II) complexes modeling the tri-nuclear copper site in multi-copper oxidases. Other researchers that have studied model complexes of multi-copper oxidases include, Cole *et al.* 1996. Kataoka *et al.* 2009 studied the mechanism of the four-electron reduction of dioxygen by a multi-copper oxidase.

The title compound C_50_H_69_Br_2_Cu_3_N_10_O_6_ is a mimic of the multi-copper enzymes such as ascorbate oxidase which is a tri-nuclear copper complex that is engaged in dioxygen (O_2_) activation and subsequent substrate oxidation (Solomon *et al.* 1996). The complex consists of two copper (II) cations bridged by a bromide anion and is thus a model for the type III site and one distorted 4-coordinate copper center and thus a model for the type II site. Taken together this comprises a model for the multi-copper oxidases which consists of a type III and type II site in close proximity. In the present case the distances from type II model center, Cu1, to the two Cu's (Cu2 and Cu3) comprising a model for the type III site are respectively 5.915 (1) and 6.565 (1) Å. The type II copper center Cu(1) is considerably distorted from square planar coordination geometry with the dihedral angle between the two chelate planes being 69.21 (7)°. The two remaining Cu's are both square pyramidal with the bridging Br in the apical position in each coordination sphere (τ values are 0.0297 (2) and 0.0386 (2), respectively [Addison *et al.*, 1984]).

There are two molecules of *N*,*N*-dimethylformamide (DMF) present as solvent molecules, one of which is disordered over two equivalent conformations with occupancies of 0.603 (5) and 0.397 (5). The amine H's are involved in both inter- and intramolecular hydrogen bonding interactions with the Br and O's of the copper cluster as well as the O of the ordered DMF.

### Experimental

The synthesis of *N*,*N*-bis(3-aminopropyl)-ethylenediamine-bis(salicylaldimine) was accomplished by adding a solution of (5 g, 30.52 mmol) *N*,*N*-bis(3-aminopropyl)-ethylene-di-amine in 20 ml methanol drop-wise to the solution of (7.45 g, 61.04 mmol) salicylaldehyde. The mixture was refluxed overnight while stirring with a magnetic stirrer. Then the reaction mixture was evaporated under reduced pressure. An oily orange product was obtained which later solidified into yellow compound.

Synthesis of the complex C_50_H_70_Br_2_Cu_3_N_10_O_6_ was achieved by adding a solution of (0.97 g, 6.76 mmol) CuBr in a mixture of 20 ml methanol and 25 ml ethanol to a solution of (1.17 g, 3.06 mmol) *N*,*N*-bis(3-aminopropyl)-ethylenediamine-bis(salicylaldimine) in 20 ml of CH_2_Cl_2_ drop-wise while stirring. The reaction time was 24 h after which the reaction mixture was evaporated under reduced pressure. Dark greenish solids were obtained. These solids were then dissolved in DMF, filtered and layered with diethyl ether for crystallization. X-ray diffraction quality crystals were obtained.

### Refinement

H atoms were placed in geometrically idealized positions and constrained to ride on their parent atoms with a C—H distances of 0.93 to 0.99 Å and N—H distances of 0.93 Å and *U*_iso_(H) = 1.2*U*_eq_(C, N).

### Crystal data

**Table:**

[Cu_3_Br(C_22_H_28_N_4_O_2_)_2_]Br·2C_3_H_7_NO	*D*_x_ = 1.457 Mg m^−^^3^
*M**_r_* = 1257.60	Mo *K*α radiation, λ = 0.71073 Å
Hexagonal, *P*6_1_	Cell parameters from 28991 reflections
Hall symbol: P 61	θ = 4.6–34.8°
*a* = 18.7450 (2) Å	µ = 2.55 mm^−^^1^
*c* = 28.2531 (5) Å	*T* = 200 K
*V* = 8597.4 (2) Å^3^	Hexagonal prism, dark green-brown
*Z* = 6	0.51 × 0.42 × 0.38 mm
*F*(000) = 3870	

### Data collection

**Table:**

Oxford Diffraction Gemini R diffractometer	11660 independent reflections
Radiation source: Enhance (Mo) X-ray Source	8463 reflections with *I* > 2σ(*I*)
graphite	*R*_int_ = 0.070
Detector resolution: 10.5081 pixels mm^-1^	θ_max_ = 26.4°, θ_min_ = 4.6°
φ and ω scans	*h* = −23→23
Absorption correction: multi-scan (*CrysAlis RED*; Oxford Diffraction, 2009)	*k* = −23→23
*T*_min_ = 0.782, *T*_max_ = 1.000	*l* = −35→35
95983 measured reflections	

### Refinement

**Table:**

Refinement on *F*^2^	Secondary atom site location: difference Fourier map
Least-squares matrix: full	Hydrogen site location: inferred from neighbouring sites
*R*[*F*^2^ > 2σ(*F*^2^)] = 0.030	H-atom parameters constrained
*wR*(*F*^2^) = 0.062	*w* = 1/[σ^2^(*F*_o_^2^) + (0.033*P*)^2^] where *P* = (*F*_o_^2^ + 2*F*_c_^2^)/3
*S* = 0.91	(Δ/σ)_max_ = 0.001
11660 reflections	Δρ_max_ = 0.39 e Å^−^^3^
662 parameters	Δρ_min_ = −0.26 e Å^−^^3^
85 restraints	Absolute structure: Flack (1983), 5710 Friedel pairs
Primary atom site location: structure-invariant direct methods	Flack parameter: 0.007 (5)

### Special details

**Table:**

Geometry. All e.s.d.'s (except the e.s.d. in the dihedral angle between two l.s. planes) are estimated using the full covariance matrix. The cell e.s.d.'s are taken into account individually in the estimation of e.s.d.'s in distances, angles and torsion angles; correlations between e.s.d.'s in cell parameters are only used when they are defined by crystal symmetry. An approximate (isotropic) treatment of cell e.s.d.'s is used for estimating e.s.d.'s involving l.s. planes.
Refinement. Refinement of *F*^2^ against ALL reflections. The weighted *R*-factor *wR* and goodness of fit *S* are based on *F*^2^, conventional *R*-factors *R* are based on *F*, with *F* set to zero for negative *F*^2^. The threshold expression of *F*^2^ > σ(*F*^2^) is used only for calculating *R*-factors(gt) *etc*. and is not relevant to the choice of reflections for refinement. *R*-factors based on *F*^2^ are statistically about twice as large as those based on *F*, and *R*- factors based on ALL data will be even larger.

### Fractional atomic coordinates and isotropic or equivalent isotropic displacement parameters (Å^2^)

**Table:**

	*x*	*y*	*z*	*U*_iso_*/*U*_eq_	Occ. (<1)
Br1	0.89066 (2)	0.14678 (2)	0.176552 (13)	0.03897 (10)	
Br2	0.74921 (3)	0.14478 (2)	−0.044701 (14)	0.04604 (11)	
Cu1	0.61482 (3)	0.32849 (3)	0.135041 (15)	0.03419 (11)	
Cu2	0.78094 (3)	0.13386 (3)	0.107128 (15)	0.03145 (11)	
Cu3	0.89767 (2)	0.27075 (3)	0.235668 (15)	0.03223 (11)	
O1A	0.52044 (15)	0.26481 (16)	0.17344 (9)	0.0477 (7)	
O2A	0.69414 (14)	0.11884 (14)	0.15022 (8)	0.0366 (6)	
O1B	0.68241 (15)	0.41100 (15)	0.09096 (9)	0.0404 (6)	
O2B	0.91309 (14)	0.34740 (15)	0.18618 (8)	0.0354 (6)	
N1A	0.57558 (18)	0.24489 (18)	0.08448 (10)	0.0335 (7)	
N2A	0.81810 (16)	0.25796 (17)	0.10297 (10)	0.0310 (7)	
H2AB	0.8436	0.2810	0.1316	0.037*	
N3A	0.86311 (18)	0.16184 (19)	0.05401 (10)	0.0369 (7)	
H3AB	0.8358	0.1603	0.0262	0.044*	
N4A	0.7301 (2)	0.01341 (18)	0.09708 (10)	0.0373 (7)	
N1B	0.67956 (18)	0.39345 (18)	0.19003 (11)	0.0366 (7)	
N2B	0.77206 (16)	0.23279 (17)	0.23541 (10)	0.0316 (7)	
H2BB	0.7489	0.2025	0.2080	0.038*	
N3B	0.86762 (18)	0.19830 (18)	0.29376 (11)	0.0369 (7)	
H3BB	0.8682	0.2306	0.3189	0.044*	
N4B	1.01824 (18)	0.32127 (19)	0.24628 (10)	0.0340 (7)	
C1A	0.4522 (2)	0.1967 (2)	0.16353 (14)	0.0379 (9)	
C2A	0.3885 (3)	0.1640 (3)	0.19581 (16)	0.0483 (11)	
H2AA	0.3952	0.1912	0.2252	0.058*	
C3A	0.3148 (3)	0.0923 (3)	0.18639 (18)	0.0561 (12)	
H3AA	0.2719	0.0717	0.2093	0.067*	
C4A	0.3029 (3)	0.0503 (3)	0.14385 (18)	0.0548 (12)	
H4AA	0.2526	0.0010	0.1374	0.066*	
C5A	0.3655 (2)	0.0818 (2)	0.11174 (16)	0.0470 (11)	
H5AA	0.3579	0.0534	0.0827	0.056*	
C6A	0.4402 (2)	0.1539 (2)	0.11959 (14)	0.0371 (9)	
C7A	0.5029 (2)	0.1788 (2)	0.08426 (14)	0.0392 (10)	
H7AA	0.4903	0.1434	0.0577	0.047*	
C8A	0.6293 (2)	0.2537 (3)	0.04497 (13)	0.0388 (9)	
H8AA	0.6594	0.3119	0.0347	0.047*	
H8AB	0.5954	0.2200	0.0180	0.047*	
C9A	0.6915 (2)	0.2267 (2)	0.05827 (13)	0.0359 (9)	
H9AA	0.6608	0.1684	0.0685	0.043*	
H9AB	0.7233	0.2296	0.0296	0.043*	
C10A	0.7520 (2)	0.2777 (2)	0.09742 (13)	0.0309 (8)	
H10A	0.7766	0.3370	0.0899	0.037*	
H10B	0.7218	0.2672	0.1277	0.037*	
C11A	0.8822 (2)	0.2975 (2)	0.06630 (14)	0.0388 (9)	
H11A	0.9216	0.3551	0.0754	0.047*	
H11B	0.8567	0.2980	0.0357	0.047*	
C12A	0.9270 (2)	0.2491 (2)	0.06150 (14)	0.0401 (9)	
H12A	0.9654	0.2697	0.0343	0.048*	
H12B	0.9590	0.2548	0.0905	0.048*	
C13A	0.8980 (3)	0.1078 (3)	0.04635 (15)	0.0497 (11)	
H13A	0.9301	0.1095	0.0746	0.060*	
H13B	0.9361	0.1284	0.0190	0.060*	
C14A	0.8327 (3)	0.0214 (3)	0.03727 (15)	0.0538 (12)	
H14A	0.8588	−0.0101	0.0259	0.065*	
H14B	0.7962	0.0210	0.0118	0.065*	
C15A	0.7814 (3)	−0.0209 (3)	0.08029 (15)	0.0511 (11)	
H15A	0.7450	−0.0800	0.0729	0.061*	
H15B	0.8185	−0.0172	0.1063	0.061*	
C16A	0.6539 (2)	−0.0364 (2)	0.10388 (13)	0.0384 (9)	
H16A	0.6337	−0.0916	0.0939	0.046*	
C17A	0.5946 (2)	−0.0182 (2)	0.12504 (13)	0.0358 (8)	
C18A	0.5128 (2)	−0.0815 (2)	0.12627 (14)	0.0449 (10)	
H18A	0.4980	−0.1320	0.1107	0.054*	
C19A	0.4536 (2)	−0.0734 (3)	0.14896 (16)	0.0514 (11)	
H19A	0.3979	−0.1168	0.1487	0.062*	
C20A	0.4760 (2)	−0.0002 (3)	0.17263 (15)	0.0452 (10)	
H20A	0.4351	0.0061	0.1889	0.054*	
C21A	0.5553 (2)	0.0624 (2)	0.17298 (15)	0.0424 (10)	
H21A	0.5687	0.1115	0.1897	0.051*	
C22A	0.6180 (2)	0.0567 (2)	0.14935 (12)	0.0309 (8)	
C1B	0.7506 (2)	0.4813 (2)	0.09864 (14)	0.0383 (9)	
C2B	0.7961 (2)	0.5279 (2)	0.05984 (15)	0.0445 (10)	
H2BA	0.7775	0.5078	0.0288	0.053*	
C3B	0.8672 (3)	0.6021 (3)	0.06554 (19)	0.0562 (12)	
H3BA	0.8974	0.6316	0.0384	0.067*	
C4B	0.8959 (3)	0.6350 (3)	0.1102 (2)	0.0591 (12)	
H4BA	0.9440	0.6875	0.1139	0.071*	
C5B	0.8525 (3)	0.5892 (2)	0.14919 (18)	0.0536 (12)	
H5BA	0.8719	0.6104	0.1800	0.064*	
C6B	0.7802 (2)	0.5119 (2)	0.14443 (15)	0.0377 (9)	
C7B	0.7438 (2)	0.4664 (2)	0.18719 (14)	0.0383 (10)	
H7BA	0.7693	0.4924	0.2162	0.046*	
C8B	0.6577 (2)	0.3565 (2)	0.23718 (14)	0.0395 (9)	
H8BA	0.6001	0.3409	0.2444	0.047*	
H8BB	0.6938	0.3973	0.2610	0.047*	
C9B	0.6671 (2)	0.2805 (2)	0.24022 (13)	0.0331 (8)	
H9BA	0.6347	0.2418	0.2146	0.040*	
H9BB	0.6447	0.2525	0.2708	0.040*	
C10B	0.7567 (2)	0.3023 (2)	0.23597 (13)	0.0297 (8)	
H10C	0.7795	0.3344	0.2065	0.036*	
H10D	0.7877	0.3388	0.2628	0.036*	
C11B	0.7338 (2)	0.1768 (2)	0.27697 (14)	0.0408 (9)	
H11C	0.7345	0.2091	0.3048	0.049*	
H11D	0.6757	0.1357	0.2698	0.049*	
C12B	0.7810 (2)	0.1338 (2)	0.28765 (14)	0.0398 (9)	
H12C	0.7599	0.1007	0.3169	0.048*	
H12D	0.7757	0.0967	0.2613	0.048*	
C13B	0.9210 (3)	0.1646 (3)	0.30828 (15)	0.0494 (11)	
H13C	0.9189	0.1257	0.2839	0.059*	
H13D	0.9004	0.1340	0.3384	0.059*	
C14B	1.0095 (2)	0.2337 (3)	0.31471 (14)	0.0476 (10)	
H14C	1.0100	0.2757	0.3361	0.057*	
H14D	1.0413	0.2110	0.3302	0.057*	
C15B	1.0516 (2)	0.2746 (3)	0.26925 (14)	0.0470 (10)	
H15C	1.1108	0.3123	0.2758	0.056*	
H15D	1.0475	0.2318	0.2470	0.056*	
C16B	1.0690 (2)	0.3952 (2)	0.23404 (13)	0.0370 (9)	
H16B	1.1240	0.4169	0.2447	0.044*	
C17B	1.0528 (2)	0.4498 (2)	0.20587 (12)	0.0328 (9)	
C18B	1.1168 (2)	0.5309 (2)	0.19848 (14)	0.0428 (10)	
H18B	1.1675	0.5495	0.2147	0.051*	
C19B	1.1089 (3)	0.5836 (2)	0.16907 (17)	0.0542 (12)	
H19B	1.1534	0.6380	0.1645	0.065*	
C20B	1.0348 (3)	0.5570 (2)	0.14577 (16)	0.0486 (11)	
H20B	1.0286	0.5937	0.1252	0.058*	
C21B	0.9708 (2)	0.4791 (2)	0.15188 (14)	0.0410 (10)	
H21B	0.9206	0.4623	0.1354	0.049*	
C22B	0.9771 (2)	0.4226 (2)	0.18211 (12)	0.0321 (8)	
O1S	0.91150 (16)	0.37375 (17)	0.31116 (10)	0.0474 (7)	
N1S	1.01679 (18)	0.5040 (2)	0.32238 (12)	0.0432 (8)	
C11S	0.9451 (2)	0.4480 (3)	0.30412 (14)	0.0453 (10)	
H11E	0.9177	0.4672	0.2839	0.054*	
C12S	1.0533 (3)	0.5915 (3)	0.31260 (19)	0.0672 (14)	
H12E	1.0121	0.6019	0.2977	0.101*	
H12F	1.1003	0.6092	0.2913	0.101*	
H12G	1.0719	0.6225	0.3423	0.101*	
C13S	1.0625 (3)	0.4780 (3)	0.35186 (19)	0.0731 (14)	
H13E	1.0376	0.4180	0.3500	0.110*	
H13F	1.0612	0.4940	0.3847	0.110*	
H13G	1.1197	0.5043	0.3409	0.110*	
O2S	0.2671 (7)	0.1242 (6)	0.2966 (3)	0.107 (3)	0.603 (5)
N2S	0.2666 (6)	0.0724 (9)	0.3677 (4)	0.067 (2)	0.603 (5)
C21S	0.2957 (7)	0.1004 (6)	0.3245 (4)	0.088 (2)	0.603 (5)
H21C	0.3448	0.1008	0.3156	0.105*	0.603 (5)
C22S	0.3171 (7)	0.0563 (7)	0.3991 (4)	0.096 (3)	0.603 (5)
H22A	0.3734	0.0823	0.3867	0.143*	0.603 (5)
H22B	0.2947	−0.0033	0.4016	0.143*	0.603 (5)
H22C	0.3176	0.0789	0.4305	0.143*	0.603 (5)
C23S	0.1892 (6)	0.0658 (7)	0.3821 (5)	0.112 (3)	0.603 (5)
H23A	0.1712	0.0909	0.3580	0.167*	0.603 (5)
H23B	0.1968	0.0944	0.4124	0.167*	0.603 (5)
H23C	0.1474	0.0076	0.3858	0.167*	0.603 (5)
O2T	0.2328 (10)	0.0872 (10)	0.2965 (5)	0.107 (3)	0.397 (5)
N2T	0.2414 (11)	0.0553 (14)	0.3716 (5)	0.067 (2)	0.397 (5)
C21T	0.2120 (10)	0.0795 (9)	0.3365 (5)	0.088 (2)	0.397 (5)
H21D	0.1701	0.0920	0.3441	0.105*	0.397 (5)
C22T	0.1978 (10)	0.0412 (10)	0.4154 (6)	0.096 (3)	0.397 (5)
H22D	0.1405	0.0259	0.4090	0.143*	0.397 (5)
H22E	0.2241	0.0915	0.4346	0.143*	0.397 (5)
H22F	0.1990	−0.0036	0.4327	0.143*	0.397 (5)
C23T	0.3184 (10)	0.0576 (13)	0.3674 (8)	0.112 (3)	0.397 (5)
H23D	0.3537	0.1008	0.3450	0.167*	0.397 (5)
H23E	0.3090	0.0042	0.3560	0.167*	0.397 (5)
H23F	0.3454	0.0694	0.3984	0.167*	0.397 (5)

### Atomic displacement parameters (Å^2^)

**Table:**

	*U*^11^	*U*^22^	*U*^33^	*U*^12^	*U*^13^	*U*^23^
Br1	0.0436 (2)	0.0452 (2)	0.0341 (2)	0.02668 (19)	−0.00490 (18)	−0.00110 (18)
Br2	0.0450 (2)	0.0467 (2)	0.0418 (2)	0.0194 (2)	−0.00779 (19)	−0.00269 (19)
Cu1	0.0310 (2)	0.0342 (3)	0.0389 (3)	0.0175 (2)	−0.0050 (2)	−0.0046 (2)
Cu2	0.0293 (2)	0.0329 (2)	0.0330 (2)	0.0161 (2)	0.0025 (2)	0.0023 (2)
Cu3	0.0261 (2)	0.0359 (3)	0.0321 (2)	0.0136 (2)	−0.0004 (2)	0.0037 (2)
O1A	0.0366 (16)	0.0515 (17)	0.0465 (16)	0.0156 (14)	0.0016 (13)	−0.0051 (14)
O2A	0.0281 (14)	0.0340 (14)	0.0425 (15)	0.0116 (12)	0.0029 (11)	−0.0039 (12)
O1B	0.0407 (15)	0.0363 (15)	0.0426 (15)	0.0179 (14)	−0.0059 (12)	−0.0002 (12)
O2B	0.0262 (13)	0.0373 (15)	0.0348 (14)	0.0100 (12)	−0.0015 (11)	0.0037 (11)
N1A	0.0340 (18)	0.0340 (18)	0.0395 (19)	0.0222 (16)	−0.0054 (14)	−0.0021 (14)
N2A	0.0252 (16)	0.0350 (17)	0.0322 (16)	0.0145 (14)	−0.0003 (13)	−0.0006 (14)
N3A	0.0414 (19)	0.048 (2)	0.0290 (17)	0.0279 (17)	−0.0007 (14)	0.0055 (15)
N4A	0.045 (2)	0.0344 (18)	0.0372 (18)	0.0232 (17)	0.0011 (15)	0.0002 (14)
N1B	0.0349 (18)	0.0343 (18)	0.043 (2)	0.0190 (16)	−0.0025 (14)	−0.0029 (15)
N2B	0.0270 (15)	0.0278 (16)	0.0315 (16)	0.0072 (13)	0.0026 (13)	0.0030 (13)
N3B	0.0396 (18)	0.0419 (18)	0.0325 (17)	0.0229 (16)	−0.0014 (14)	−0.0012 (15)
N4B	0.0303 (17)	0.042 (2)	0.0282 (17)	0.0175 (16)	−0.0020 (13)	−0.0029 (14)
C1A	0.027 (2)	0.037 (2)	0.054 (3)	0.0186 (19)	−0.0086 (19)	−0.003 (2)
C2A	0.042 (3)	0.050 (3)	0.053 (3)	0.023 (2)	0.004 (2)	0.006 (2)
C3A	0.040 (3)	0.052 (3)	0.080 (4)	0.026 (2)	0.009 (2)	0.028 (3)
C4A	0.041 (3)	0.046 (3)	0.073 (3)	0.018 (2)	−0.007 (2)	0.004 (2)
C5A	0.038 (2)	0.039 (2)	0.067 (3)	0.021 (2)	−0.005 (2)	0.003 (2)
C6A	0.031 (2)	0.030 (2)	0.052 (2)	0.0168 (18)	−0.0063 (19)	0.0022 (19)
C7A	0.042 (2)	0.037 (2)	0.045 (2)	0.024 (2)	−0.012 (2)	−0.0070 (18)
C8A	0.039 (2)	0.050 (2)	0.034 (2)	0.0273 (19)	−0.0106 (18)	−0.0094 (18)
C9A	0.038 (2)	0.040 (2)	0.034 (2)	0.0232 (19)	−0.0067 (17)	−0.0078 (17)
C10A	0.0275 (19)	0.034 (2)	0.035 (2)	0.0176 (17)	0.0009 (16)	−0.0029 (17)
C11A	0.031 (2)	0.039 (2)	0.039 (2)	0.0113 (18)	0.0029 (18)	0.0072 (18)
C12A	0.026 (2)	0.050 (2)	0.041 (2)	0.0161 (19)	0.0058 (17)	0.0151 (19)
C13A	0.059 (3)	0.065 (3)	0.044 (2)	0.045 (3)	0.016 (2)	0.012 (2)
C14A	0.071 (3)	0.064 (3)	0.048 (3)	0.050 (3)	0.008 (2)	0.000 (2)
C15A	0.058 (3)	0.052 (3)	0.053 (3)	0.035 (2)	0.008 (2)	−0.003 (2)
C16A	0.044 (2)	0.028 (2)	0.042 (2)	0.016 (2)	−0.0022 (19)	−0.0006 (18)
C17A	0.032 (2)	0.038 (2)	0.036 (2)	0.0161 (18)	−0.0067 (17)	0.0022 (17)
C18A	0.041 (2)	0.031 (2)	0.049 (3)	0.0086 (19)	−0.007 (2)	−0.0006 (19)
C19A	0.027 (2)	0.048 (3)	0.064 (3)	0.007 (2)	−0.002 (2)	0.013 (2)
C20A	0.031 (2)	0.053 (3)	0.053 (3)	0.022 (2)	0.0028 (19)	0.009 (2)
C21A	0.033 (2)	0.038 (2)	0.054 (3)	0.0161 (19)	0.000 (2)	−0.003 (2)
C22A	0.027 (2)	0.030 (2)	0.030 (2)	0.0103 (17)	−0.0013 (16)	0.0080 (16)
C1B	0.036 (2)	0.036 (2)	0.051 (3)	0.024 (2)	0.001 (2)	0.002 (2)
C2B	0.039 (2)	0.046 (3)	0.051 (3)	0.023 (2)	−0.001 (2)	0.006 (2)
C3B	0.046 (3)	0.059 (3)	0.074 (3)	0.033 (2)	0.014 (3)	0.023 (3)
C4B	0.040 (2)	0.042 (3)	0.082 (4)	0.010 (2)	−0.001 (3)	0.009 (3)
C5B	0.047 (3)	0.036 (2)	0.073 (3)	0.017 (2)	−0.012 (2)	−0.003 (2)
C6B	0.027 (2)	0.032 (2)	0.056 (3)	0.0163 (18)	−0.0030 (19)	0.003 (2)
C7B	0.036 (2)	0.031 (2)	0.052 (3)	0.020 (2)	−0.0107 (19)	−0.0129 (19)
C8B	0.040 (2)	0.039 (2)	0.042 (2)	0.0210 (19)	−0.0048 (19)	−0.0127 (19)
C9B	0.0275 (19)	0.029 (2)	0.041 (2)	0.0136 (17)	0.0014 (17)	−0.0029 (17)
C10B	0.0305 (19)	0.0244 (18)	0.034 (2)	0.0140 (16)	−0.0057 (16)	−0.0006 (16)
C11B	0.035 (2)	0.040 (2)	0.044 (2)	0.0161 (19)	0.0076 (18)	0.0119 (19)
C12B	0.037 (2)	0.037 (2)	0.041 (2)	0.0156 (19)	0.0087 (18)	0.0129 (18)
C13B	0.053 (3)	0.061 (3)	0.043 (2)	0.035 (2)	0.006 (2)	0.021 (2)
C14B	0.043 (2)	0.066 (3)	0.040 (2)	0.033 (2)	−0.0040 (19)	0.007 (2)
C15B	0.030 (2)	0.060 (3)	0.049 (3)	0.021 (2)	−0.0094 (19)	0.001 (2)
C16B	0.0233 (19)	0.050 (3)	0.034 (2)	0.0159 (19)	−0.0038 (17)	−0.012 (2)
C17B	0.033 (2)	0.036 (2)	0.028 (2)	0.0171 (18)	−0.0011 (16)	−0.0096 (16)
C18B	0.035 (2)	0.041 (2)	0.047 (2)	0.015 (2)	−0.0027 (19)	−0.018 (2)
C19B	0.043 (3)	0.027 (2)	0.081 (3)	0.009 (2)	0.013 (2)	−0.002 (2)
C20B	0.051 (3)	0.031 (2)	0.063 (3)	0.020 (2)	0.002 (2)	0.000 (2)
C21B	0.033 (2)	0.039 (2)	0.050 (2)	0.0174 (19)	0.0007 (19)	−0.001 (2)
C22B	0.029 (2)	0.033 (2)	0.032 (2)	0.0141 (18)	0.0039 (17)	−0.0042 (17)
O1S	0.0400 (16)	0.0433 (18)	0.0516 (17)	0.0155 (14)	0.0030 (13)	−0.0044 (14)
N1S	0.0308 (18)	0.042 (2)	0.051 (2)	0.0142 (17)	−0.0042 (16)	−0.0107 (16)
C11S	0.035 (2)	0.057 (3)	0.046 (3)	0.025 (2)	0.002 (2)	−0.008 (2)
C12S	0.049 (3)	0.049 (3)	0.092 (4)	0.015 (2)	0.007 (3)	−0.007 (3)
C13S	0.063 (3)	0.071 (3)	0.075 (3)	0.027 (3)	−0.032 (3)	−0.019 (3)
O2S	0.121 (6)	0.112 (7)	0.082 (3)	0.055 (5)	−0.004 (4)	−0.004 (4)
N2S	0.066 (6)	0.049 (5)	0.078 (3)	0.022 (5)	0.006 (3)	0.006 (3)
C21S	0.104 (5)	0.087 (5)	0.079 (5)	0.053 (4)	−0.009 (4)	−0.012 (4)
C22S	0.099 (5)	0.083 (5)	0.106 (5)	0.047 (4)	0.009 (4)	0.029 (4)
C23S	0.105 (6)	0.108 (6)	0.119 (6)	0.050 (5)	0.018 (5)	−0.002 (5)
O2T	0.121 (6)	0.112 (7)	0.082 (3)	0.055 (5)	−0.004 (4)	−0.004 (4)
N2T	0.066 (6)	0.049 (5)	0.078 (3)	0.022 (5)	0.006 (3)	0.006 (3)
C21T	0.104 (5)	0.087 (5)	0.079 (5)	0.053 (4)	−0.009 (4)	−0.012 (4)
C22T	0.099 (5)	0.083 (5)	0.106 (5)	0.047 (4)	0.009 (4)	0.029 (4)
C23T	0.105 (6)	0.108 (6)	0.119 (6)	0.050 (5)	0.018 (5)	−0.002 (5)

### Geometric parameters (Å, °)

**Table:**

Br1—Cu2	2.7636 (6)	C21A—C22A	1.401 (5)
Br1—Cu3	2.8108 (6)	C21A—H21A	0.9500
Cu1—O1B	1.895 (3)	C1B—C2B	1.396 (5)
Cu1—O1A	1.903 (3)	C1B—C6B	1.411 (5)
Cu1—N1A	1.971 (3)	C2B—C3B	1.372 (6)
Cu1—N1B	1.973 (3)	C2B—H2BA	0.9500
Cu2—O2A	1.937 (2)	C3B—C4B	1.390 (6)
Cu2—N4A	1.984 (3)	C3B—H3BA	0.9500
Cu2—N3A	2.023 (3)	C4B—C5B	1.383 (6)
Cu2—N2A	2.071 (3)	C4B—H4BA	0.9500
Cu3—O2B	1.920 (2)	C5B—C6B	1.412 (5)
Cu3—N4B	1.989 (3)	C5B—H5BA	0.9500
Cu3—N3B	2.022 (3)	C6B—C7B	1.439 (5)
Cu3—N2B	2.092 (3)	C7B—H7BA	0.9500
O1A—C1A	1.309 (4)	C8B—C9B	1.521 (5)
O2A—C22A	1.316 (4)	C8B—H8BA	0.9900
O1B—C1B	1.316 (4)	C8B—H8BB	0.9900
O2B—C22B	1.323 (4)	C9B—C10B	1.522 (5)
N1A—C7A	1.304 (5)	C9B—H9BA	0.9900
N1A—C8A	1.457 (4)	C9B—H9BB	0.9900
N2A—C10A	1.468 (4)	C10B—H10C	0.9900
N2A—C11A	1.475 (4)	C10B—H10D	0.9900
N2A—H2AB	0.9300	C11B—C12B	1.496 (5)
N3A—C13A	1.470 (5)	C11B—H11C	0.9900
N3A—C12A	1.482 (5)	C11B—H11D	0.9900
N3A—H3AB	0.9300	C12B—H12C	0.9900
N4A—C16A	1.271 (4)	C12B—H12D	0.9900
N4A—C15A	1.478 (5)	C13B—C14B	1.521 (6)
N1B—C7B	1.296 (4)	C13B—H13C	0.9900
N1B—C8B	1.462 (5)	C13B—H13D	0.9900
N2B—C10B	1.469 (4)	C14B—C15B	1.502 (5)
N2B—C11B	1.498 (4)	C14B—H14C	0.9900
N2B—H2BB	0.9300	C14B—H14D	0.9900
N3B—C12B	1.471 (4)	C15B—H15C	0.9900
N3B—C13B	1.483 (5)	C15B—H15D	0.9900
N3B—H3BB	0.9300	C16B—C17B	1.444 (5)
N4B—C16B	1.275 (4)	C16B—H16B	0.9500
N4B—C15B	1.458 (5)	C17B—C18B	1.403 (5)
C1A—C2A	1.379 (5)	C17B—C22B	1.414 (5)
C1A—C6A	1.433 (5)	C18B—C19B	1.355 (6)
C2A—C3A	1.389 (6)	C18B—H18B	0.9500
C2A—H2AA	0.9500	C19B—C20B	1.384 (6)
C3A—C4A	1.393 (6)	C19B—H19B	0.9500
C3A—H3AA	0.9500	C20B—C21B	1.361 (5)
C4A—C5A	1.362 (6)	C20B—H20B	0.9500
C4A—H4AA	0.9500	C21B—C22B	1.410 (5)
C5A—C6A	1.394 (5)	C21B—H21B	0.9500
C5A—H5AA	0.9500	O1S—C11S	1.223 (5)
C6A—C7A	1.431 (5)	N1S—C11S	1.328 (5)
C7A—H7AA	0.9500	N1S—C13S	1.443 (5)
C8A—C9A	1.532 (5)	N1S—C12S	1.454 (5)
C8A—H8AA	0.9900	C11S—H11E	0.9500
C8A—H8AB	0.9900	C12S—H12E	0.9800
C9A—C10A	1.530 (5)	C12S—H12F	0.9800
C9A—H9AA	0.9900	C12S—H12G	0.9800
C9A—H9AB	0.9900	C13S—H13E	0.9800
C10A—H10A	0.9900	C13S—H13F	0.9800
C10A—H10B	0.9900	C13S—H13G	0.9800
C11A—C12A	1.518 (5)	O2S—C21S	1.160 (11)
C11A—H11A	0.9900	N2S—C21S	1.334 (11)
C11A—H11B	0.9900	N2S—C22S	1.435 (11)
C12A—H12A	0.9900	N2S—C23S	1.450 (13)
C12A—H12B	0.9900	C21S—H21C	0.9500
C13A—C14A	1.485 (6)	C22S—H22A	0.9800
C13A—H13A	0.9900	C22S—H22B	0.9800
C13A—H13B	0.9900	C22S—H22C	0.9800
C14A—C15A	1.506 (6)	C23S—H23A	0.9800
C14A—H14A	0.9900	C23S—H23B	0.9800
C14A—H14B	0.9900	C23S—H23C	0.9800
C15A—H15A	0.9900	O2T—C21T	1.179 (14)
C15A—H15B	0.9900	N2T—C21T	1.320 (13)
C16A—C17A	1.444 (5)	N2T—C23T	1.428 (17)
C16A—H16A	0.9500	N2T—C22T	1.435 (15)
C17A—C18A	1.395 (5)	C21T—H21D	0.9500
C17A—C22A	1.422 (5)	C22T—H22D	0.9800
C18A—C19A	1.355 (6)	C22T—H22E	0.9800
C18A—H18A	0.9500	C22T—H22F	0.9800
C19A—C20A	1.388 (6)	C23T—H23D	0.9800
C19A—H19A	0.9500	C23T—H23E	0.9800
C20A—C21A	1.357 (5)	C23T—H23F	0.9800
C20A—H20A	0.9500		
			
Cu2—Br1—Cu3	102.672 (17)	C18A—C19A—C20A	118.6 (4)
O1B—Cu1—O1A	156.87 (11)	C18A—C19A—H19A	120.7
O1B—Cu1—N1A	90.23 (12)	C20A—C19A—H19A	120.7
O1A—Cu1—N1A	93.11 (12)	C21A—C20A—C19A	121.2 (4)
O1B—Cu1—N1B	93.44 (12)	C21A—C20A—H20A	119.4
O1A—Cu1—N1B	91.73 (12)	C19A—C20A—H20A	119.4
N1A—Cu1—N1B	158.65 (12)	C20A—C21A—C22A	121.9 (4)
O2A—Cu2—N4A	91.93 (11)	C20A—C21A—H21A	119.0
O2A—Cu2—N3A	168.39 (11)	C22A—C21A—H21A	119.0
N4A—Cu2—N3A	93.40 (12)	O2A—C22A—C21A	120.3 (3)
O2A—Cu2—N2A	89.16 (10)	O2A—C22A—C17A	123.0 (3)
N4A—Cu2—N2A	166.07 (12)	C21A—C22A—C17A	116.7 (3)
N3A—Cu2—N2A	83.21 (12)	O1B—C1B—C2B	118.7 (4)
O2A—Cu2—Br1	95.79 (8)	O1B—C1B—C6B	123.1 (3)
N4A—Cu2—Br1	96.58 (9)	C2B—C1B—C6B	118.2 (3)
N3A—Cu2—Br1	93.84 (9)	C3B—C2B—C1B	121.5 (4)
N2A—Cu2—Br1	97.13 (8)	C3B—C2B—H2BA	119.3
O2B—Cu3—N4B	92.49 (11)	C1B—C2B—H2BA	119.3
O2B—Cu3—N3B	168.65 (11)	C2B—C3B—C4B	121.4 (4)
N4B—Cu3—N3B	93.79 (12)	C2B—C3B—H3BA	119.3
O2B—Cu3—N2B	88.55 (10)	C4B—C3B—H3BA	119.3
N4B—Cu3—N2B	168.69 (12)	C5B—C4B—C3B	118.0 (4)
N3B—Cu3—N2B	83.46 (11)	C5B—C4B—H4BA	121.0
O2B—Cu3—Br1	96.50 (7)	C3B—C4B—H4BA	121.0
N4B—Cu3—Br1	93.18 (9)	C4B—C5B—C6B	121.8 (4)
N3B—Cu3—Br1	92.58 (9)	C4B—C5B—H5BA	119.1
N2B—Cu3—Br1	97.90 (8)	C6B—C5B—H5BA	119.1
C1A—O1A—Cu1	129.7 (2)	C1B—C6B—C5B	119.0 (4)
C22A—O2A—Cu2	125.3 (2)	C1B—C6B—C7B	123.7 (3)
C1B—O1B—Cu1	128.8 (2)	C5B—C6B—C7B	117.1 (4)
C22B—O2B—Cu3	125.3 (2)	N1B—C7B—C6B	126.3 (3)
C7A—N1A—C8A	116.0 (3)	N1B—C7B—H7BA	116.8
C7A—N1A—Cu1	123.8 (3)	C6B—C7B—H7BA	116.8
C8A—N1A—Cu1	120.2 (2)	N1B—C8B—C9B	111.1 (3)
C10A—N2A—C11A	112.1 (3)	N1B—C8B—H8BA	109.4
C10A—N2A—Cu2	115.9 (2)	C9B—C8B—H8BA	109.4
C11A—N2A—Cu2	109.6 (2)	N1B—C8B—H8BB	109.4
C10A—N2A—H2AB	106.2	C9B—C8B—H8BB	109.4
C11A—N2A—H2AB	106.2	H8BA—C8B—H8BB	108.0
Cu2—N2A—H2AB	106.2	C8B—C9B—C10B	111.9 (3)
C13A—N3A—C12A	112.9 (3)	C8B—C9B—H9BA	109.2
C13A—N3A—Cu2	118.3 (2)	C10B—C9B—H9BA	109.2
C12A—N3A—Cu2	105.7 (2)	C8B—C9B—H9BB	109.2
C13A—N3A—H3AB	106.4	C10B—C9B—H9BB	109.2
C12A—N3A—H3AB	106.4	H9BA—C9B—H9BB	107.9
Cu2—N3A—H3AB	106.4	N2B—C10B—C9B	116.3 (3)
C16A—N4A—C15A	117.8 (3)	N2B—C10B—H10C	108.2
C16A—N4A—Cu2	122.6 (2)	C9B—C10B—H10C	108.2
C15A—N4A—Cu2	119.6 (3)	N2B—C10B—H10D	108.2
C7B—N1B—C8B	116.7 (3)	C9B—C10B—H10D	108.2
C7B—N1B—Cu1	124.2 (3)	H10C—C10B—H10D	107.4
C8B—N1B—Cu1	119.0 (2)	C12B—C11B—N2B	109.3 (3)
C10B—N2B—C11B	112.1 (3)	C12B—C11B—H11C	109.8
C10B—N2B—Cu3	112.6 (2)	N2B—C11B—H11C	109.8
C11B—N2B—Cu3	107.4 (2)	C12B—C11B—H11D	109.8
C10B—N2B—H2BB	108.2	N2B—C11B—H11D	109.8
C11B—N2B—H2BB	108.2	H11C—C11B—H11D	108.3
Cu3—N2B—H2BB	108.2	N3B—C12B—C11B	106.8 (3)
C12B—N3B—C13B	112.6 (3)	N3B—C12B—H12C	110.4
C12B—N3B—Cu3	106.2 (2)	C11B—C12B—H12C	110.4
C13B—N3B—Cu3	119.3 (2)	N3B—C12B—H12D	110.4
C12B—N3B—H3BB	105.9	C11B—C12B—H12D	110.4
C13B—N3B—H3BB	105.9	H12C—C12B—H12D	108.6
Cu3—N3B—H3BB	105.9	N3B—C13B—C14B	110.6 (3)
C16B—N4B—C15B	117.5 (3)	N3B—C13B—H13C	109.5
C16B—N4B—Cu3	122.0 (2)	C14B—C13B—H13C	109.5
C15B—N4B—Cu3	120.5 (2)	N3B—C13B—H13D	109.5
O1A—C1A—C2A	119.9 (4)	C14B—C13B—H13D	109.5
O1A—C1A—C6A	122.3 (3)	H13C—C13B—H13D	108.1
C2A—C1A—C6A	117.7 (3)	C15B—C14B—C13B	113.8 (3)
C1A—C2A—C3A	121.7 (4)	C15B—C14B—H14C	108.8
C1A—C2A—H2AA	119.2	C13B—C14B—H14C	108.8
C3A—C2A—H2AA	119.2	C15B—C14B—H14D	108.8
C2A—C3A—C4A	120.9 (4)	C13B—C14B—H14D	108.8
C2A—C3A—H3AA	119.6	H14C—C14B—H14D	107.7
C4A—C3A—H3AA	119.6	N4B—C15B—C14B	114.4 (3)
C5A—C4A—C3A	118.0 (4)	N4B—C15B—H15C	108.7
C5A—C4A—H4AA	121.0	C14B—C15B—H15C	108.7
C3A—C4A—H4AA	121.0	N4B—C15B—H15D	108.7
C4A—C5A—C6A	123.0 (4)	C14B—C15B—H15D	108.7
C4A—C5A—H5AA	118.5	H15C—C15B—H15D	107.6
C6A—C5A—H5AA	118.5	N4B—C16B—C17B	127.8 (3)
C5A—C6A—C7A	118.0 (4)	N4B—C16B—H16B	116.1
C5A—C6A—C1A	118.7 (4)	C17B—C16B—H16B	116.1
C7A—C6A—C1A	123.2 (3)	C18B—C17B—C22B	118.7 (3)
N1A—C7A—C6A	127.2 (4)	C18B—C17B—C16B	118.8 (3)
N1A—C7A—H7AA	116.4	C22B—C17B—C16B	122.3 (3)
C6A—C7A—H7AA	116.4	C19B—C18B—C17B	122.3 (4)
N1A—C8A—C9A	111.9 (3)	C19B—C18B—H18B	118.8
N1A—C8A—H8AA	109.2	C17B—C18B—H18B	118.8
C9A—C8A—H8AA	109.2	C18B—C19B—C20B	119.0 (4)
N1A—C8A—H8AB	109.2	C18B—C19B—H19B	120.5
C9A—C8A—H8AB	109.2	C20B—C19B—H19B	120.5
H8AA—C8A—H8AB	107.9	C21B—C20B—C19B	120.9 (4)
C10A—C9A—C8A	114.8 (3)	C21B—C20B—H20B	119.5
C10A—C9A—H9AA	108.6	C19B—C20B—H20B	119.5
C8A—C9A—H9AA	108.6	C20B—C21B—C22B	121.5 (4)
C10A—C9A—H9AB	108.6	C20B—C21B—H21B	119.3
C8A—C9A—H9AB	108.6	C22B—C21B—H21B	119.3
H9AA—C9A—H9AB	107.5	O2B—C22B—C21B	118.7 (3)
N2A—C10A—C9A	112.0 (3)	O2B—C22B—C17B	123.7 (3)
N2A—C10A—H10A	109.2	C21B—C22B—C17B	117.6 (3)
C9A—C10A—H10A	109.2	C11S—N1S—C13S	119.7 (4)
N2A—C10A—H10B	109.2	C11S—N1S—C12S	122.5 (4)
C9A—C10A—H10B	109.2	C13S—N1S—C12S	117.9 (3)
H10A—C10A—H10B	107.9	O1S—C11S—N1S	125.3 (4)
N2A—C11A—C12A	108.5 (3)	O1S—C11S—H11E	117.4
N2A—C11A—H11A	110.0	N1S—C11S—H11E	117.4
C12A—C11A—H11A	110.0	N1S—C12S—H12E	109.5
N2A—C11A—H11B	110.0	N1S—C12S—H12F	109.5
C12A—C11A—H11B	110.0	H12E—C12S—H12F	109.5
H11A—C11A—H11B	108.4	N1S—C12S—H12G	109.5
N3A—C12A—C11A	106.8 (3)	H12E—C12S—H12G	109.5
N3A—C12A—H12A	110.4	H12F—C12S—H12G	109.5
C11A—C12A—H12A	110.4	N1S—C13S—H13E	109.5
N3A—C12A—H12B	110.4	N1S—C13S—H13F	109.5
C11A—C12A—H12B	110.4	H13E—C13S—H13F	109.5
H12A—C12A—H12B	108.6	N1S—C13S—H13G	109.5
N3A—C13A—C14A	111.6 (3)	H13E—C13S—H13G	109.5
N3A—C13A—H13A	109.3	H13F—C13S—H13G	109.5
C14A—C13A—H13A	109.3	C21S—N2S—C22S	117.7 (10)
N3A—C13A—H13B	109.3	C21S—N2S—C23S	119.1 (10)
C14A—C13A—H13B	109.3	C22S—N2S—C23S	123.0 (10)
H13A—C13A—H13B	108.0	O2S—C21S—N2S	126.6 (12)
C13A—C14A—C15A	113.4 (4)	O2S—C21S—H21C	116.7
C13A—C14A—H14A	108.9	N2S—C21S—H21C	116.7
C15A—C14A—H14A	108.9	C21T—N2T—C23T	121.2 (15)
C13A—C14A—H14B	108.9	C21T—N2T—C22T	114.6 (15)
C15A—C14A—H14B	108.9	C23T—N2T—C22T	123.4 (14)
H14A—C14A—H14B	107.7	O2T—C21T—N2T	126.3 (17)
N4A—C15A—C14A	114.4 (3)	O2T—C21T—H21D	116.8
N4A—C15A—H15A	108.7	N2T—C21T—H21D	116.8
C14A—C15A—H15A	108.7	N2T—C22T—H22D	109.5
N4A—C15A—H15B	108.7	N2T—C22T—H22E	109.5
C14A—C15A—H15B	108.7	H22D—C22T—H22E	109.5
H15A—C15A—H15B	107.6	N2T—C22T—H22F	109.5
N4A—C16A—C17A	127.1 (3)	H22D—C22T—H22F	109.5
N4A—C16A—H16A	116.4	H22E—C22T—H22F	109.5
C17A—C16A—H16A	116.4	N2T—C23T—H23D	109.5
C18A—C17A—C22A	119.6 (3)	N2T—C23T—H23E	109.5
C18A—C17A—C16A	117.3 (3)	H23D—C23T—H23E	109.5
C22A—C17A—C16A	122.7 (3)	N2T—C23T—H23F	109.5
C19A—C18A—C17A	122.0 (4)	H23D—C23T—H23F	109.5
C19A—C18A—H18A	119.0	H23E—C23T—H23F	109.5
C17A—C18A—H18A	119.0		
			
Cu3—Br1—Cu2—O2A	−52.80 (7)	C8A—N1A—C7A—C6A	177.2 (3)
Cu3—Br1—Cu2—N4A	−145.43 (9)	Cu1—N1A—C7A—C6A	−1.2 (5)
Cu3—Br1—Cu2—N3A	120.70 (9)	C5A—C6A—C7A—N1A	178.1 (3)
Cu3—Br1—Cu2—N2A	37.07 (8)	C1A—C6A—C7A—N1A	−5.5 (6)
Cu2—Br1—Cu3—O2B	−47.97 (7)	C7A—N1A—C8A—C9A	−99.0 (4)
Cu2—Br1—Cu3—N4B	−140.84 (9)	Cu1—N1A—C8A—C9A	79.5 (3)
Cu2—Br1—Cu3—N3B	125.21 (8)	N1A—C8A—C9A—C10A	−62.8 (4)
Cu2—Br1—Cu3—N2B	41.46 (8)	C11A—N2A—C10A—C9A	78.5 (4)
O1B—Cu1—O1A—C1A	89.5 (4)	Cu2—N2A—C10A—C9A	−48.5 (3)
N1A—Cu1—O1A—C1A	−8.4 (3)	C8A—C9A—C10A—N2A	−170.5 (3)
N1B—Cu1—O1A—C1A	−167.6 (3)	C10A—N2A—C11A—C12A	−157.2 (3)
N4A—Cu2—O2A—C22A	−28.0 (3)	Cu2—N2A—C11A—C12A	−26.9 (3)
N3A—Cu2—O2A—C22A	89.4 (6)	C13A—N3A—C12A—C11A	177.1 (3)
N2A—Cu2—O2A—C22A	138.1 (3)	Cu2—N3A—C12A—C11A	−52.1 (3)
Br1—Cu2—O2A—C22A	−124.8 (3)	N2A—C11A—C12A—N3A	52.6 (4)
O1A—Cu1—O1B—C1B	107.1 (4)	C12A—N3A—C13A—C14A	−178.0 (3)
N1A—Cu1—O1B—C1B	−154.4 (3)	Cu2—N3A—C13A—C14A	57.9 (4)
N1B—Cu1—O1B—C1B	4.6 (3)	N3A—C13A—C14A—C15A	−70.2 (4)
N4B—Cu3—O2B—C22B	−26.6 (3)	C16A—N4A—C15A—C14A	128.7 (4)
N3B—Cu3—O2B—C22B	97.0 (6)	Cu2—N4A—C15A—C14A	−50.3 (4)
N2B—Cu3—O2B—C22B	142.1 (3)	C13A—C14A—C15A—N4A	66.5 (5)
Br1—Cu3—O2B—C22B	−120.1 (2)	C15A—N4A—C16A—C17A	172.5 (4)
O1B—Cu1—N1A—C7A	−150.6 (3)	Cu2—N4A—C16A—C17A	−8.5 (5)
O1A—Cu1—N1A—C7A	6.5 (3)	N4A—C16A—C17A—C18A	176.1 (4)
N1B—Cu1—N1A—C7A	109.3 (4)	N4A—C16A—C17A—C22A	−12.0 (6)
O1B—Cu1—N1A—C8A	31.0 (2)	C22A—C17A—C18A—C19A	1.8 (6)
O1A—Cu1—N1A—C8A	−171.9 (2)	C16A—C17A—C18A—C19A	174.1 (4)
N1B—Cu1—N1A—C8A	−69.1 (4)	C17A—C18A—C19A—C20A	−1.6 (6)
O2A—Cu2—N2A—C10A	−44.3 (2)	C18A—C19A—C20A—C21A	0.4 (6)
N4A—Cu2—N2A—C10A	50.3 (6)	C19A—C20A—C21A—C22A	0.5 (6)
N3A—Cu2—N2A—C10A	126.9 (2)	Cu2—O2A—C22A—C21A	−162.7 (3)
Br1—Cu2—N2A—C10A	−140.1 (2)	Cu2—O2A—C22A—C17A	17.2 (5)
O2A—Cu2—N2A—C11A	−172.5 (2)	C20A—C21A—C22A—O2A	179.6 (3)
N4A—Cu2—N2A—C11A	−77.9 (5)	C20A—C21A—C22A—C17A	−0.3 (6)
N3A—Cu2—N2A—C11A	−1.3 (2)	C18A—C17A—C22A—O2A	179.2 (3)
Br1—Cu2—N2A—C11A	91.8 (2)	C16A—C17A—C22A—O2A	7.5 (5)
O2A—Cu2—N3A—C13A	−153.7 (5)	C18A—C17A—C22A—C21A	−0.9 (5)
N4A—Cu2—N3A—C13A	−36.5 (3)	C16A—C17A—C22A—C21A	−172.6 (3)
N2A—Cu2—N3A—C13A	157.1 (3)	Cu1—O1B—C1B—C2B	171.7 (2)
Br1—Cu2—N3A—C13A	60.3 (3)	Cu1—O1B—C1B—C6B	−8.0 (5)
O2A—Cu2—N3A—C12A	78.7 (6)	O1B—C1B—C2B—C3B	179.3 (3)
N4A—Cu2—N3A—C12A	−164.1 (2)	C6B—C1B—C2B—C3B	−1.0 (5)
N2A—Cu2—N3A—C12A	29.5 (2)	C1B—C2B—C3B—C4B	−1.6 (6)
Br1—Cu2—N3A—C12A	−67.3 (2)	C2B—C3B—C4B—C5B	2.6 (6)
O2A—Cu2—N4A—C16A	23.2 (3)	C3B—C4B—C5B—C6B	−1.2 (6)
N3A—Cu2—N4A—C16A	−146.5 (3)	O1B—C1B—C6B—C5B	−177.9 (3)
N2A—Cu2—N4A—C16A	−71.1 (6)	C2B—C1B—C6B—C5B	2.3 (5)
Br1—Cu2—N4A—C16A	119.3 (3)	O1B—C1B—C6B—C7B	6.1 (6)
O2A—Cu2—N4A—C15A	−157.8 (3)	C2B—C1B—C6B—C7B	−173.7 (3)
N3A—Cu2—N4A—C15A	32.5 (3)	C4B—C5B—C6B—C1B	−1.3 (6)
N2A—Cu2—N4A—C15A	107.9 (5)	C4B—C5B—C6B—C7B	175.0 (4)
Br1—Cu2—N4A—C15A	−61.7 (3)	C8B—N1B—C7B—C6B	174.9 (3)
O1B—Cu1—N1B—C7B	0.2 (3)	Cu1—N1B—C7B—C6B	−1.5 (5)
O1A—Cu1—N1B—C7B	−157.3 (3)	C1B—C6B—C7B—N1B	−1.2 (6)
N1A—Cu1—N1B—C7B	99.6 (4)	C5B—C6B—C7B—N1B	−177.2 (3)
O1B—Cu1—N1B—C8B	−176.2 (2)	C7B—N1B—C8B—C9B	−114.7 (3)
O1A—Cu1—N1B—C8B	26.3 (3)	Cu1—N1B—C8B—C9B	61.9 (3)
N1A—Cu1—N1B—C8B	−76.7 (4)	N1B—C8B—C9B—C10B	66.7 (4)
O2B—Cu3—N2B—C10B	−47.5 (2)	C11B—N2B—C10B—C9B	−53.5 (4)
N4B—Cu3—N2B—C10B	47.9 (7)	Cu3—N2B—C10B—C9B	−174.8 (2)
N3B—Cu3—N2B—C10B	124.4 (2)	C8B—C9B—C10B—N2B	−176.2 (3)
Br1—Cu3—N2B—C10B	−143.9 (2)	C10B—N2B—C11B—C12B	−153.5 (3)
O2B—Cu3—N2B—C11B	−171.4 (2)	Cu3—N2B—C11B—C12B	−29.3 (3)
N4B—Cu3—N2B—C11B	−76.0 (6)	C13B—N3B—C12B—C11B	175.5 (3)
N3B—Cu3—N2B—C11B	0.5 (2)	Cu3—N3B—C12B—C11B	−52.1 (3)
Br1—Cu3—N2B—C11B	92.3 (2)	N2B—C11B—C12B—N3B	54.5 (4)
O2B—Cu3—N3B—C12B	73.8 (6)	C12B—N3B—C13B—C14B	−179.3 (3)
N4B—Cu3—N3B—C12B	−162.7 (2)	Cu3—N3B—C13B—C14B	55.2 (4)
N2B—Cu3—N3B—C12B	28.3 (2)	N3B—C13B—C14B—C15B	−69.3 (4)
Br1—Cu3—N3B—C12B	−69.4 (2)	C16B—N4B—C15B—C14B	129.3 (4)
O2B—Cu3—N3B—C13B	−157.7 (5)	Cu3—N4B—C15B—C14B	−49.8 (4)
N4B—Cu3—N3B—C13B	−34.2 (3)	C13B—C14B—C15B—N4B	67.2 (5)
N2B—Cu3—N3B—C13B	156.8 (3)	C15B—N4B—C16B—C17B	171.6 (4)
Br1—Cu3—N3B—C13B	59.2 (3)	Cu3—N4B—C16B—C17B	−9.3 (5)
O2B—Cu3—N4B—C16B	22.2 (3)	N4B—C16B—C17B—C18B	176.7 (3)
N3B—Cu3—N4B—C16B	−148.3 (3)	N4B—C16B—C17B—C22B	−9.0 (6)
N2B—Cu3—N4B—C16B	−72.8 (7)	C22B—C17B—C18B—C19B	−0.7 (5)
Br1—Cu3—N4B—C16B	118.9 (3)	C16B—C17B—C18B—C19B	173.9 (4)
O2B—Cu3—N4B—C15B	−158.7 (3)	C17B—C18B—C19B—C20B	0.6 (6)
N3B—Cu3—N4B—C15B	30.7 (3)	C18B—C19B—C20B—C21B	−0.4 (6)
N2B—Cu3—N4B—C15B	106.3 (6)	C19B—C20B—C21B—C22B	0.1 (6)
Br1—Cu3—N4B—C15B	−62.1 (3)	Cu3—O2B—C22B—C21B	−163.7 (2)
Cu1—O1A—C1A—C2A	−175.0 (3)	Cu3—O2B—C22B—C17B	17.3 (5)
Cu1—O1A—C1A—C6A	4.6 (5)	C20B—C21B—C22B—O2B	−179.2 (3)
O1A—C1A—C2A—C3A	179.2 (3)	C20B—C21B—C22B—C17B	−0.2 (5)
C6A—C1A—C2A—C3A	−0.3 (5)	C18B—C17B—C22B—O2B	179.4 (3)
C1A—C2A—C3A—C4A	0.6 (6)	C16B—C17B—C22B—O2B	5.1 (5)
C2A—C3A—C4A—C5A	−0.4 (6)	C18B—C17B—C22B—C21B	0.4 (5)
C3A—C4A—C5A—C6A	−0.1 (6)	C16B—C17B—C22B—C21B	−173.9 (3)
C4A—C5A—C6A—C7A	177.0 (4)	C13S—N1S—C11S—O1S	2.2 (6)
C4A—C5A—C6A—C1A	0.4 (6)	C12S—N1S—C11S—O1S	−179.9 (4)
O1A—C1A—C6A—C5A	−179.7 (3)	C22S—N2S—C21S—O2S	−171.6 (12)
C2A—C1A—C6A—C5A	−0.2 (5)	C23S—N2S—C21S—O2S	4(2)
O1A—C1A—C6A—C7A	3.9 (5)	C23T—N2T—C21T—O2T	18 (3)
C2A—C1A—C6A—C7A	−176.5 (3)	C22T—N2T—C21T—O2T	−172.4 (18)

### Hydrogen-bond geometry (Å, °)

**Table:**

*D*—H···*A*	*D*—H	H···*A*	*D*···*A*	*D*—H···*A*
N2A—H2AB···O2B	0.93	2.00	2.919 (4)	170.
N3A—H3AB···Br2	0.93	2.50	3.429 (3)	174.
N2B—H2BB···O2A	0.93	2.14	3.061 (4)	172.
N3B—H3BB···O1S	0.93	2.39	3.005 (4)	123.
